# Post-tuberculosis chronic pulmonary aspergillosis: An emerging public health concern

**DOI:** 10.1371/journal.ppat.1008742

**Published:** 2020-08-20

**Authors:** Felix Bongomin

**Affiliations:** Department of Medical Microbiology and Immunology, Faculty of Medicine, Gulu University, Gulu, Uganda; Vallabhbhai Patel Chest Institute, INDIA

## Introduction

Tuberculosis (TB) caused by *Mycobacterium tuberculosis* complex is the leading cause of death and diseases from a single infectious agent and continues to be a major global public health problem [1]. However, with early diagnosis and commencement of an appropriate treatment regimen, an estimated 58 (53–64) million lives globally have been saved from TB between 2000 and 2018 [1,2].

## Are these lives saved forever?

In the last few decades, there has been a renewed interest in the study of the long-term outcomes of patients previously treated for TB [3,4]. In a recent meta-analysis, persons previously treated for TB were found to be 2 to 5 times more likely to die compared to the general population or matched controls [5]. The excess mortality among these individuals has been attributed to the increased risk of recurrent TB, as well as related communicable and noncommunicable diseases [3,5–7].

Pulmonary TB (PTB), the most common form of TB, causes extensive structural lung changes in more than two-thirds of the patients that persists after treatment despite microbiological cure of the active disease [8–10]. These residual changes can be categorized into parenchymal, airway disease, pleural/chest wall, vascular, and mediastinal pathologies, collectively referred to as post-TB lung disease (PTBLD) [3,8]. Infectious complications, including chronic pulmonary aspergillosis (CPA), is common in PTBLD [3,4].

## How common is CPA after treated PTB?

CPA is a progressive respiratory syndrome that largely occur in immunocompetent or subtly immunocompromised individuals with underlying structural lung diseases, most commonly treated TB [11]. Residual cavities remain in between 20% to 40% of lungs of patients following treatment for PTB [12,13]. Cavitation and ectatic lesions in PTBLD allows saprophytic colonisation and growth of *Aspergillus* species following inhalation of infectious spores from the environment. This leads to expansion of the existing or creation of new cavities with associated parenchymal and pleural damage [11]. A complex mixture of *Aspergillus* hyphae, tissue debris, inflammatory cells, and mucin known as a fungal ball (aspergilloma) may form in these cavities [11].

The significance of treated PTB in the pathogenesis of CPA was established by a large cohort study across over 50 clinics in Great Britain in the mid- to late 1960s [14]. In this landmark study, 544 patients who had a persistent cavity of at least 2.5 cm in diameter and negative *M*. *tuberculosis* bacilli in their sputum for at least a year were studied. About 25% of the patients had positive *Aspergillus* precipitins (i.e., precipitating immunoglobulin M [IgM] and immunoglobulin G [IgG] antibodies) in their serum, 14% had aspergillomas, and 10% had precipitins without evidence of an aspergilloma. Three years later, 34% had positive *Aspergillus* precipitins, 22% had aspergillomas, and 14% had precipitins alone. In more recent studies among patients previously or currently being treated for TB, up to 39% of whom had residual cavities, the prevalence of CPA ranged between 6.3% and 13.7% [13,15,16]. Among patients with CPA, treated PTB is the primary underlying respiratory condition in 17%–93% of the cases [12,17].

In high-burden TB countries, the proportion of CPA with TB as underlying condition is higher than low–TB-burden countries [12,17,18]. However, the precise burden of post-TB CPA remains unknown. Based on a deterministic model, the global burden of post-TB CPA was estimated at 1.2 million cases annually [19]. In this model, the prevalence rate ranged from <1 case per 100,000 population in the United States (low TB burden) compared to 42.9 per 100,000 in Nigeria (high TB burden) [19]. A similar mathematical modelling was performed to estimate the burden of CPA-complicating TB in India [20]. In this study, the annual incidence of CPA was estimated at between 27,000 and 170,000 cases given the very high TB incidence rate of 2.1 million cases [20].

It is important to note that most patients with CPA have multiple underlying lung conditions [12]. Other important non-TB underlying conditions include chronic obstructive pulmonary disease (COPD), fibrocystic sarcoidosis, pneumothorax, and other [12,21].

## Why is CPA misdiagnosed as PTB?

CPA presents with chronic productive cough, systemic symptoms (weight loss, fatigue, fevers, etc.), haemoptysis, and chest pain [22]. These symptoms are clinically indistinguishable from those of PTB. Furthermore, progressive cavitation, fibrosis, and pleural thickening seen in confirmed CPA are similar to radiological features observed in PTB, PTBLD, and in relapse of PTB [3,8,14]. PTB constitutes over 85% of the global TB burden; however, only 65% of these patients are bacteriologically confirmed cases [23]. The non-bacteriologically confirmed cases likely are a mixture of patients, including those with false-negative PTB and those with PTBLD, including CPA. In a single study among Nigerians, 13 of the 17 patients with CPA were being treated for “smear-negative PTB” [15]. In addition, the lack of awareness and low index of suspicion for CPA among clinicians, as well as the non-availability of essential diagnostics for CPA, are associated with misdiagnosis of CPA [24]. Other differential diagnoses such as chronic cavitary pulmonary histoplasmosis [25], coccidioidomycosis, paracoccidioidomycosis in endemic areas, non-tuberculous mycobacterial (NTM) infection, and other cavitary lung diseases should be considered and evaluated if indicated.

## How is CPA differentiated from PTB and PTBLD?

Given the striking similarities in the clinical and radiological manifestations of these diseases, more specific microbiological and serological tests are required to make a definitive diagnosis [22,26]. *Aspergillus*-specific IgG is particularly sensitive and is positive in over 90% of patients with CPA [11,27–29]. The diagnosis of CPA requires a combination of characteristics: one or more cavities with or without a fungal ball present or nodules on thoracic imaging, direct evidence of *Aspergillus* infection (microscopy or culture from biopsy) or an immunological response to *Aspergillus* spp., and exclusion of alternative diagnoses, all present for at least 3 months [22,26]. Isolation of *Aspergillus* spp. is time consuming, has low sensitivity, and may represent colonization or contamination. Therefore, *Aspergillus* serology is the cornerstone for CPA diagnosis. Introduction of a cheap and rapid point-of-care *Aspergillus* IgG/IgM lateral flow assay with a sensitivity and specificity of 91.6% (86%–95.4%) and 98.0% (94.2%–99.6%), respectively, has made the diagnosis of CPA possible even in resource-constrained settings [24,27]. PTB can usually be excluded through routine sputum microscopy or culture or by use of the rapid molecular assay (GeneXpert) [30,31]. However, NTM infection cannot be diagnosed with *M*. *tuberculosis* GeneXpert. NTM is a cause of smear-positive/*M*. *tuberculosis* GeneXpert-negative mycobacterial infection with a very high risk of concurrent or precedent CPA development [32]. CPA patients with NTM have a particularly poorer prognosis [32]. There is currently no guidance on the identification and management of PTBLD [3]. PTBLD is a subject of further research, and management is based on expert opinion.

## Can PTB and CPA coexist?

Both CPA and PTB are caused by the opportunistic respiratory pathogens, *Aspergillus* spp. and *M*. *tuberculosis*, respectively [1,11]. However, co-isolation is uncommon, and colonisation is suggested in most cases [33]. A systematic review and meta-analysis of cross-sectional studies from Asia and Africa showed an *Aspergillus* co-infection rate of 15.4% (95% CI: 11.4–20.5) among patients with PTB [34].

In one study, co-isolation of *Aspergillus* in respiratory samples of patients with confirmed *M*. *tuberculosis* was achieved in 50 out of 140 (35.7%) patients, of whom 92% had underlying lung conditions, 35 (70%) had positive *Aspergillus* IgG, and 3 (6%) were confirmed to have CPA [33]. Among HIV-TB co-infected Ugandans, 9% of the patients had a positive *Aspergillus* IgG at the end of their TB treatment [35]. However, full diagnostic work-up for CPA was not performed. CPA-PTB-*Klebsiella pneumoniae* triple co-infection has also been described in a patient with poorly controlled diabetes mellitus [36]. Two clinical studies demonstrated a CPA prevalence of 13.7% (of 124 HIV-negative TB patients) [13] and 8.7% (of 208 153-HIV positive TB patients) patients in the last month of their TB treatment [15].

Concurrent treatment of CPA and TB is very challenging due to the significant drug-drug interactions between anti-TB agents and the triazoles. Rifampicin markedly reduces exposures to itraconazole to sub-therapeutic levels, and hence co-administration should be avoided.

## How is post-TB CPA treated?

The management of post-TB CPA is the same as that for any underlying lung disease such as COPD or sarcoidosis and depends on the radiological phenotype of CPA [22]. An incidental finding of an asymptomatic *Aspergillus* nodule may not necessitate therapy; a simple aspergilloma occurring in a well-circumscribed cavity without extensive destruction of surrounding lung tissue is best managed surgically. Meanwhile chronic cavitary pulmonary aspergillosis (CCPA) and chronic fibrosing pulmonary aspergillosis (CFPA)—characterised by multiple enlargement of existent or creation of new cavities and extensive destructive fibrotic pleuro-parenchymal changes—respectively are the most common forms of aspergillosis and always require antifungal management [22,37].

Long-term oral antifungals with itraconazole at a dose of 400 mg/day or voriconazole at a dose of 400 mg/day administered for at least 6 months is the recommended first-line therapy for CPA associated with improvement in quality of life, relief of symptoms, and retardation of disease progression [38,39]. Newer generation triazoles with better pharmacodynamics and pharmacokinetic profiles, namely, posaconazole and isavuconazole, are alternative azoles for the salvage therapy for patients who are intolerant or have *Aspergillus* spp. isolates resistant to the first-line agents [40–42]. Azole resistance is an increasingly important challenge in the management of patients with CPA. Resistance can develop in-host during treatment (patient route) or alternatively through exposure to azole fungicides in the environment (environmental route) [43]. In cases of complete intolerance or pan-azole resistance, short courses of intravenous amphotericin B or an echinocandin has been used to treat CPA with an overall response rate of about 61% (95% CI: 52%–70%) [44].

Antifungal treatment improves survival. However, survival rates vary significantly among published studies. Reported survival rates are 58%–93% at 1 year of follow-up, 17.5%–85% at 5 years of follow-up, and 30%–50% at 10 years of follow-up [45–49]. In a selected group of patients with CPA, weekly subcutaneous injections of interferon gamma (IFNγ) has been shown to improve disease control (reduced frequency of exacerbation and hospitalisation) and also helps with bacterial clearance [50]. Several factors have been reported to affect mortality, including by underlying pulmonary disease, advanced age, NTM infection, quality of life scores, and serum albumin levels [49].

In addition to conventional antifungal treatment, pulmonary rehabilitation has been shown to improve symptoms and quality of life of patients with PTBLD, including those with post-TB CPA [3]. At the moment, it is unclear whether all post-TB patients should be screened for CPA or whether genetic risk profiling may be of help. Management occurs in the context of a multidisciplinary setting, including chest physicians, radiologists, infectious diseases physicians, chest physiotherapist, occupational therapist, pharmacists, and nurses.

## Conclusion

The relationship between CPA and TB is well established. CPA complicates previously treated TB due to the residual structural lesions. About one-third of patients previously treated for PTB will have residual lung cavities. Specific serological and microbiological tests are mandatory to differentiate CPA from active PTB disease, recurrent PTB, and PTBLD.
